# Down‐regulation of Shh in the hair follicles of mice during chemotherapy‐induced hair loss is mediated by the JAK/STAT1 signaling pathway

**DOI:** 10.1002/2211-5463.70160

**Published:** 2025-11-20

**Authors:** Ruifang Fan, Jingling Huang, Xiang Lin, Tingjiao Lan, Yuli Tang, Ling Sun, Guixuan Zhou

**Affiliations:** ^1^ College of Biological Science and Engineering Fuzhou University China

**Keywords:** chemotherapy‐induced alopecia, hair follicles, JAK/STAT1 signaling pathway, Shh‐expressing cells

## Abstract

Chemotherapy‐induced alopecia (CIA) is a major unresolved adverse effect in clinical oncology. We have previously shown that the Sonic hedgehog (Shh) signaling pathway is targeted by cyclophosphamide (CYP) treatment, but the detailed mechanism by which this chemotherapy drug induces alopecia still remains largely unknown. To answer this question, in the present study, we used Shh‐GFP^+/−^ mice and analyzed Shh‐expressing cells (Shh^+^ cells) in hair follicles at different times post‐CYP treatment. Through flow cytometry assays, we showed that Shh^+^ cells decreased significantly after CYP treatment. To investigate the molecular events involved in this decrease, we carried out gene set enrichment analysis of RNA sequencing data of Shh^+^ cells, which revealed that the expression levels of most Janus‐activated kinase/signal transducer and activator of transcription 1 (JAK/STAT1) signaling pathway‐related genes were upregulated compared to the controls. Furthermore, through a chromatin immunoprecipitation assay, we showed that STAT1 could bind to the promoter of the Shh gene during CIA in hair follicles and the binding strength increased upon CYP treatment. Treatment with JAK inhibitors also rescued hair loss and upregulated Shh expression in mice with CIA, further supporting the role of the JAK/STAT1 signaling pathway in the regulation of Shh during CIA. Taken together, our results provide new insights into the molecular mechanisms of CIA.

## Graphical abstract

We found that during chemotherapy‐induced alopecia (CIA), Sonic hedgehog (Shh) expression significantly decreased in hair follicle Shh^+^ cells, whereas the Janus‐activated kinase/signal transducer and activator of transcription 1 (JAK/STAT1) signaling pathway was markedly activated. This pathway directly inhibits Shh expression through STAT1 binding to the Shh gene promoter. Using JAK kinase inhibitors to block this pathway upregulated Shh expression and promoted hair follicle recovery in CIA.

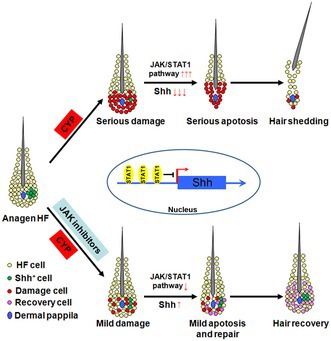

**Abbreviations**
ChIP: chromatin immunoprecipitation
CIA: chemotherapy‐induced alopecia
CYP: cyclophosphamide
DEGs: differentially expressed genes
GSEA: gene set enrichment analysis
H&E: hemotoxylin and eosin
HFSC: hair follicle stem cell
JAK: Janus‐activated kinase
KEGG: Kyoto Encyclopedia of Genes and Genomes
PBS: phosphate‐buffered saline
PFA: paraformaldehyde
Shh: sonic hedgehog
STAT1: signal transducer and activator of transcription 1

Chemotherapy‐induced alopecia (CIA) is the most common dermatological side effect in patients undergoing chemotherapy [1, 2]. Patients usually suffer great emotional impact when their hair begins to fall out [3]. In the past decades, the preventative treatments for some adverse effects of cancer therapy have improved, but the CIA remains a great challenge to cure [4]. This is mainly a result of the complex mechanism of CIA. Some signaling pathways, such as the p53 signaling pathway and the apoptosis pathway, have been well demonstrated to regulate CIA [5, 6]. However, no efficient treatment for CIA had been developed based on these findings.

In previous studies, we have demonstrated that the Sonic hedgehog (Shh) signaling pathway is an important target of chemo‐drugs in both a feather CIA model and the classic mouse CIA model [7, 8]. However, how chemo‐drugs suppress Shh remains largely unknown. To explore this problem, Shh‐GFP transgenic mice were used in the present study. The Shh‐expressing cells (Shh^+^ cells) in hair follicles before or post cyclophosphamide (CYP) treatment were isolated and sorted by flow cytometry, and the next‐generation RNA sequencing was conducted to reveal the global changes in gene expression in Shh‐expressing cells at different times along with CYP treatment. The findings of this study may provide new insights into the mechanism of CIA.

## Materials and methods

### Experimental animals and procedures

The Shhgfpcre mouse strain (B6.Cg‐Shh^tm1(EGFP/cre)Cjt^/J, JAX stock #005622) [9] was purchased from the Jackson Laboratory (Bar Harbor, ME, USA), and hybridized with wild‐type C57BL/6 mice. Genotyping was conducted according to the Jax Protocol #28097 and PCR primers are shown in Table S1. Heterozygotes were used in this study with reference to Shh‐GFP^+/−^ mice. Wild‐type C57BL/6 mice were purchased from Shanghai Jihui Laboratory Animal Care Co., Ltd (Shanghai, China).

The mouse CIA model was established as described previously [10]. Briefly, hairs in the back skin of 7–8‐week‐old mice were depilated by wax to induce anagen, and CYP (Meilunbio, Dalian, China) was intraperitonally injected in mice 9 days after depilation at a dose of 150 mg·kg^−1^ to induce hair loss. For the CIA rescue assay, Janus‐activated kinase (JAK) inhibitors, including AG490 (Beyotime, Shanghai, China), ruxolitinib (Bidepharm, Shanghai, China) and tofacitinib (Rhawn, Shanghai, China), were topically treated on the dorsal skin of mice for 5 days (from day 7 to day 11 after depilation, once a day) at different doses modified from previous researches [11, 12], respectively. CYP was intraperitonally injected in mice 1 h after the treatment of JAK inhibitors on day 9. The mice were euthanized by cervical dislocation after anesthesia with sodium pentobarbital (30 mg·kg^−1^). Skin samples were taken at designated times and used for the different experiments. All experimental protocols were approved by the Ethics Committee of Fuzhou University (approval number: 2022‐SG‐025).

### Histological analysis and immunostaining

For hemotoxylin and eosin (H&E) staining, mouse skin samples were taken at day 9 after depilation before CYP treatment (referred to as day 9 + 0 or Control) or at different days after CYP treatment (referred to as day 9 + 1 to day 9 + 11). Skin samples were fixed using 4% paraformaldehyde (PFA), dehydrated and embedded in paraffin. Eight‐μm sections were prepared for staining according to the standard protocol. For immunohistochemistry staining, paraffin sections were prepared, an anti‐p53 primary antibody (#2524; CST, Danvers, MA, USA) and a 3,3′‐diaminobenzidine staining kit (ZSBio, Beijing, China) were used. For immunofluorescence staining, cryosections were prepared and incubated with an anti‐activated caspase‐3 primary antibody (AF835; R&D Systems, Minneapolis, MN, USA) at 4 °C overnight. After incubation with a secondary antibody coupled with Cy3, sections were counter‐stained with 4′,6‐diamidino‐2‐phenylindole [1 μg·mL^−1^ in phosphate‐buffered saline (PBS)]. For a whole‐mount view of the hair follicles of Shh‐GFP^+/−^ mice, skin samples were cut into small pieces, fixed by 4% PFA, and photographed under a microscope (Leica, Wetzlar, Germany).

### Flow cytometry assay and cell sorting

To isolate Shh‐GFP cells from hair follicles, the back skins of mice were dissected and subjected to chemical and mechanical digestion according to a protocol modified from a previous report [13]. Briefly, skins were dissected and the subcutaneous fats were removed by sharp forceps before incubation in 0.1% collagenase I (BBI, Shanghai, China) in Hank's buffer with the dermis side down for 15 min at 37 °C. After washing with PBS three times, the skins were incubated in 0.5% trypsin (BBI) + EDTA for another 20 min at 37 °C with the dermis side down. The dermis side of the skin was then scraped with a dull scalpel to release cells in the hair bulb. The cell suspensions were filtered using a 74‐μm cell strainer and centrifugated at 800 ***g*** for 8 min. Cells were washed with PBS three times, resuspended in PBS and subjected to a flow cytometry assay. The percentage of GFP^+^ cells was calculated and GFP cells were sorted. Because it is difficult to obtain enough single‐sex Shh‐GFP^+/−^ mice for the experiment as a result of the weak reproductive capacity, a pool of 16 Shh‐GFP^+/−^ mice was used for flow cytometry assay and cell sorting, comprising five mice (two males and three females) in the control group, five mice (two males and three females) in the CYP 12 h group and six mice (three males and three females) in the CYP 24 h group. Deviation as a result of gender differences could not be ignored in this study.

### RNA sequencing and data analysis

Total RNAs of the Shh‐GFP cells were isolated using a RNA isolater Reagent (Vazyme, Nanjing, China) and quality was monitored using a Model 2100 Bioanalyzer (Agilent, Santa Clara, CA, USA) and sequencing with an Illumina NovaSeq 6000 RNA sequencing platform (Novogene, Beijing, China). The RNA‐sequencing data were mapped to the mouse genome assembly GRCm39 using hisat2 [14]. Differentially expressed genes (DEGs) defined by two‐fold expression change and a false discovery rate of *P* < 0.05 were calculated using deseq2 [15]. The DEGs were subjected to Kyoto Encyclopedia of Genes and Genomes (KEGG) (https://www.genome.jp/kegg) or gene set enrichment analysis (GSEA) [16]. Among the 16 Shh‐GFP^+/−^ mice used in the flow cytometry assay, sorting of Shh‐GFP^+/−^ cells failed in four mice, and RNA library construction failed in another three mice because of the poor quality of RNA. As a result, Shh‐GFP^+/−^ cells from nine Shh‐GFP^+/−^ mice were used for RNA sequencing, with one male and two female mice in the control group, three female mice in the CYP 12 h group, and two males and one female mice in the CYP 24 h group. Deviation as a result of gender differences could not be ignored in this study.

### Chromatin immunoprecipitation (ChIP) assay

A ChIP assay was conducted using a ChIP‐IT High Sensitivity kit (ActiveMotif, Carlsbad, CA, USA) in accordance with the manufaturer's instructions. Briefly, skin samples were cross‐linked with 1% PFA at room temperature for 30 min, homogenized, and sonicated. The lysate was then incubated with 5 μL of rabbit anti‐signal transducer and activator of transcription 1 (STAT) antibody (CST) or normal rabbit IgG (BBI) at 4 °C overnight. The samples were incubated with A + G agarose to pull down the antibody‐protein‐DNA complex the next day. DNA was isolated from the complex after reversing cross‐link and proteinase K digesting and used for PCR. The primers are shown in Table S1.

### Real‐time quantitative PCR analysis

Mouse skin samples were collected at designated times, and total RNAs were isolated using RNA isolater Reagent (Vazyme). Reverse transcription was performed using a HiScript II 1st Strand cDNA Synthesis Kit (Vazyme). PCR was performed in a LightCycler96 real‐time PCR machine (Roche, Basel, Switzerland) using a SYBR green qPCR Mix kit (Biosharp, Beijing, China).

### Statistical analysis

All experiments were repeated at least three times and the results are shown as the mean ± SEM. A two‐tailed Student's *t*‐test was used to calculate the *P*‐value. *P* < 0.05 was considered statistically significant.

## Results

### Shh‐GFP ^+/−^ mice undergone CIA after CYP treatment

**Fig. 1:**
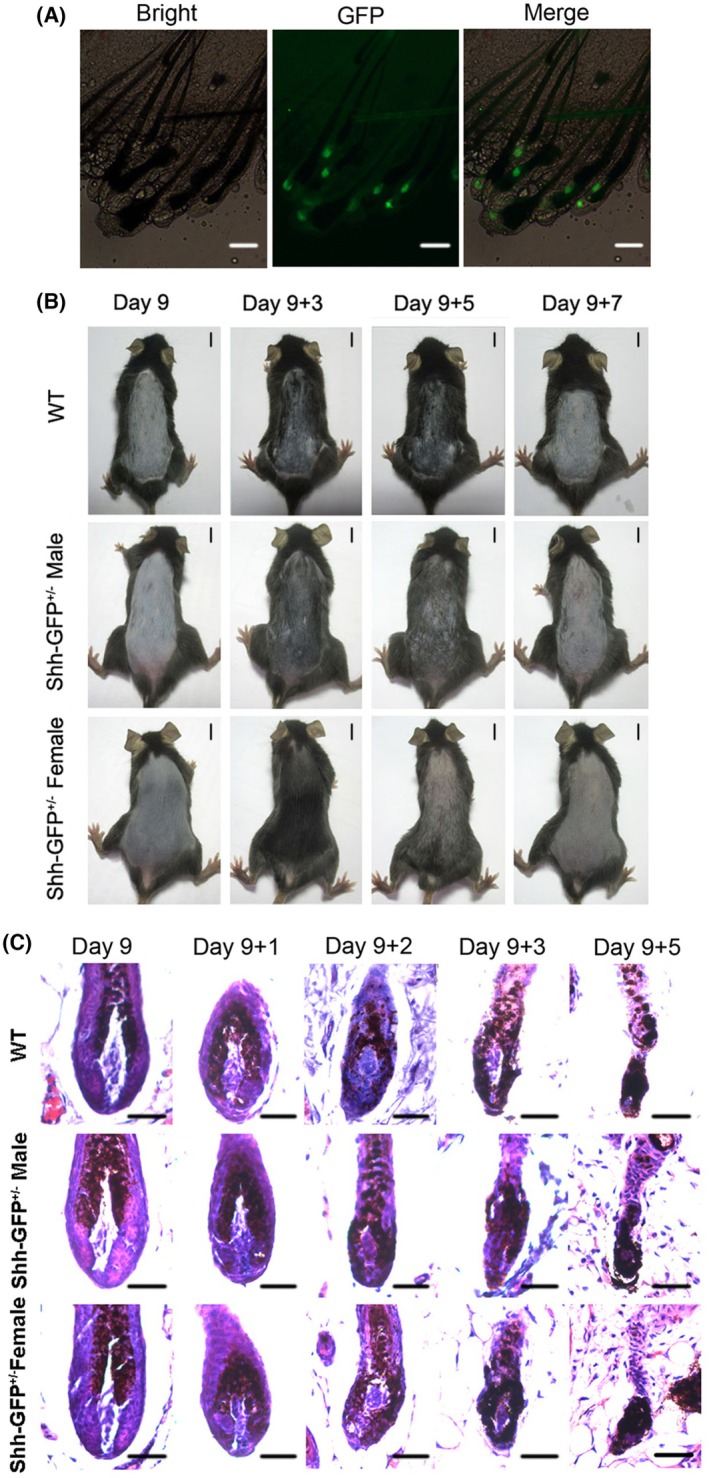
Shh‐GFP^+/−^ mice undergo CIA. (A) Whole‐mount view of Shh‐GFP^+/−^ mouse skin with anagen hair follicles under a fluorescence microscope. GFP cells represent Shh‐expressing cells (scale bar = 100 μm). (B) Hair loss occurred in wild‐type (male, *n* = 5) and Shh‐GFP^+/−^ mice (male and female, *n* = 3 for each gender) after CYP injection (scale bar = 1 cm). (C) H&E staining of hair follicles in wild‐type and Shh‐GFP^+/−^ mice at different days after CYP treatment (scale bar = 50 μm).

All transgenic mice were subjected to genotyping first and the heterozygotes (Fig. S1) were taken for the follow‐up experiments. A whole‐mount view of back skin samples containing anagen hair follicles (9 days after depilation) of the transgenic mice showed the asymmetric location of GFP cells in the matrix of hair follicles (Fig. 1A), which was in accordance with the specific expression pattern of Shh in hair follicles [17]. Following the injection of CYP, both male and female Shh‐GFP^+/−^ mice underwent CIA, as well as wild‐type mice, and almost all of the hair follicles of back skin fell out at 7 days after CYP treatment (Fig. 1B). H&E staining revealed that the hair follicles were damaged by CYP and significantly atrophied follicles were observed 2 days after treatment in both wild‐type and Shh‐GFP^+/−^ mice (Fig. 1C). The changes in morphology of hair follicle were similar between wild‐type and Shh‐GFP^+/−^ mice, as well as between male and female Shh‐GFP^+/−^ mice (Fig. 1C).

### Shh^+^ cells of hair follicles decrease during CIA

**Fig. 2:**
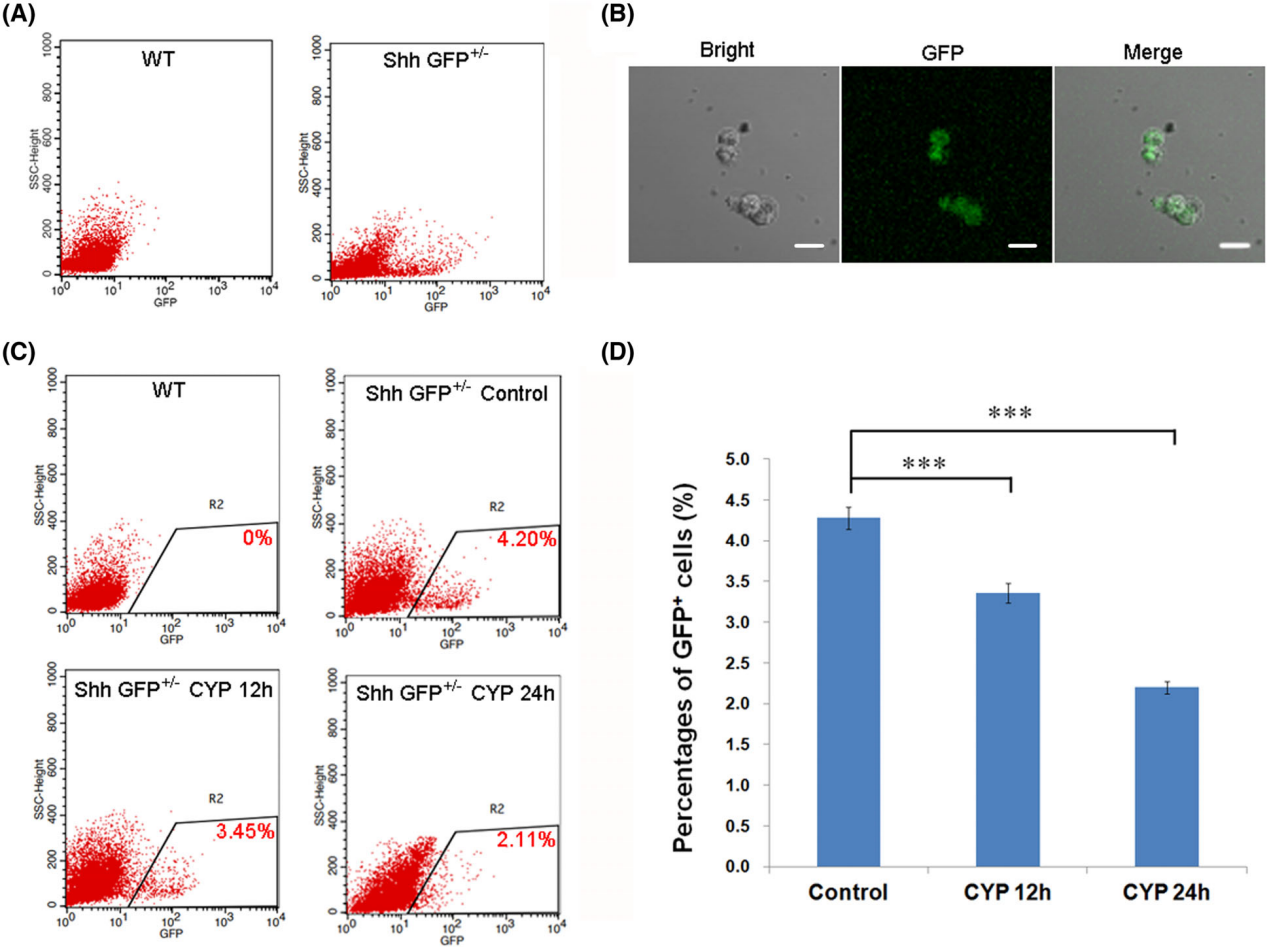
Shh‐expressing cells decreased during CIA. (A) Flow cytometry analysis of hair follicles isolated from wild‐type and Shh‐GFP^+/−^ mice. Note that a subgroup of cells was observed in Shh‐GFP^+/−^ mice, distinct from wild‐type mice. (B) The subgroup cells were sorted and photographed under a fluorescence microscope and the GFP signal was confirmed (scale bar = 10 μm). (C) Representative results of the flow cytometry of hair follicle cells from wild‐type and Shh‐GFP^+/−^ mouse at different times post‐CYP treatment. Gates were set more conservatively to minimize the capture of non‐GFP cells. (D) Changes in the percentages of GFP^+^ cells in hair follicles of Shh‐GFP^+/−^ mouse at different times post‐CYP injection. The percentages of GFP^+^ cells at different times post‐CYP injection were compared with the control group, respectively. The results are shown as the mean ± SEM. A two‐tailed Student's *t*‐test was used to calculate the *P*‐value. Control group, *n* = 5; CYP 12 h group, *n* = 5; CYP 24 h group, *n* = 6. ***P < 0.001.

To explore the influence of CYP on Shh^+^ cells in hair follicles, cells of hair bulbs were isolated from the Shh‐GFP^+/−^ mice. Flow cytometry assay revealed that a subgroup of cells displaying a stronger GFP signal in Shh‐GFP^+/−^ mice but not in wild‐type mice (Fig. 2A). The GFP signal was confirmed in cells sorted from this subgroup by fluorescence microscopy (Fig. 2B), suggesting that cells in this subgroup were Shh^+^ cells. The changes in Shh^+^ cells in hair follicles at different times along with CYP treatment were further investigated, and the results showed that the percentage of Shh^+^ cells was significantly decreased at 12 and 24 h after CYP injection, from over 4% in the control group to almost 2% in CYP 24 h group (Fig. 2C,D).

### Global changes in gene expression in Shh^+^ cells during CIA

**Fig. 3:**
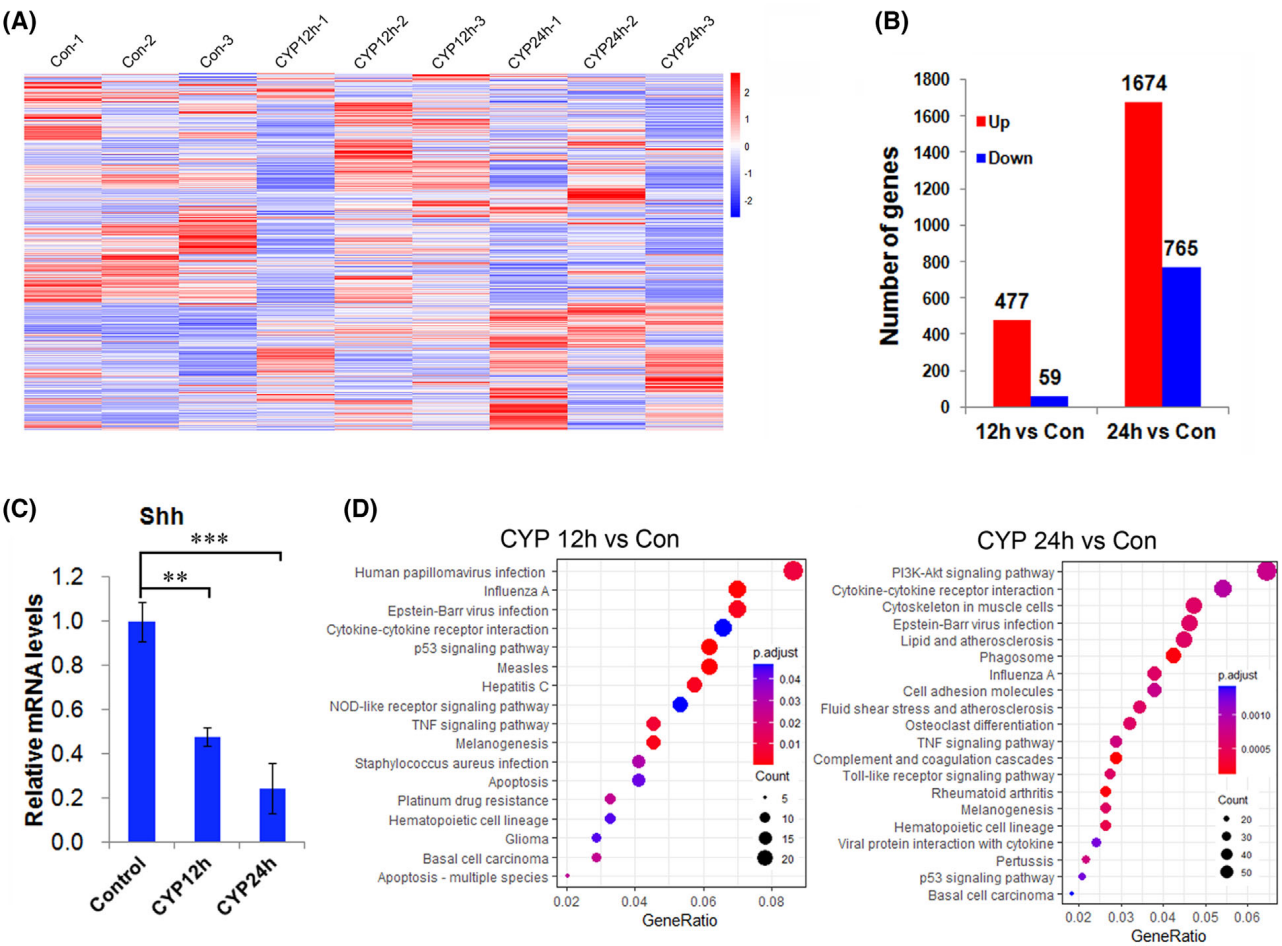
Global changes in gene expression of Shh‐expressing in hair follicles during CIA. (A) Heat map of the RNA sequencing data; *n* = 3 for each group. (B) Differentially expressed genes of CYP 12 h and CYP24h group compared to the control group, respectively. (C) Changes in mRNA level of Shh in Shh‐expressing cells at different times post‐CYP treatment. Data obtained from RNA sequencing. The mRNA levels of Shh at different times post‐CYP injection were compared with the control group, respectively. The results are shown as the mean ± SEM. A two‐tailed Student's *t*‐test was used to calculate the *P*‐value (*n* = 3; **P < 0.01; ***P < 0.001). (D) Top 20 signaling pathways enriched in CYP 12 h and CYP24h group compared to the control group, respectively. Note there was only a total of 17 significant pathways enriched in the CYP 12 h group compared to the control group.

To investigate the molecular events in Shh^+^ cells of hair follicles in CIA mice, GFP cells sorted from hair follicles of Shh‐GFP^+/−^ mice in different groups were used for RNA sequencing (raw data deposited in GSE277257). Over 25 000 genes were detectable, and the heatmap of the expressing genes is shown in Fig. 3A. Among these genes, 536 were differentially expressed in the CYP 12 h group compared to the control group, with 477 genes up‐regulated and 59 genes down‐regulated. In total, 2439 genes were differentially expressed in the CYP 24 h group compared to the control group, with 1674 genes up‐regulated and 765 genes down‐regulated (Fig. 3B; Tables S2 and S3). Among these differentially expressed genes, the expression of Shh significantly decreased in response to CYP treatment (Fig. 3C), which was in accordance with our previous finding [8].

KEGG pathway enrichment analysis revealed that 17 and 56 signaling pathways were significantly disturbed in Shh^+^ cells at 12 and 24 h after CYP injection, respectively (Fig. 3D; Tables S4 and S5). The p53 signaling pathway and the apoptosis pathway were both on the two lists of drug‐perturbed pathways at 12 and 24 h after drug injection, suggesting that these two signaling pathways play important roles in Shh^+^ cells in response to drug treatment.

**Fig. 4:**
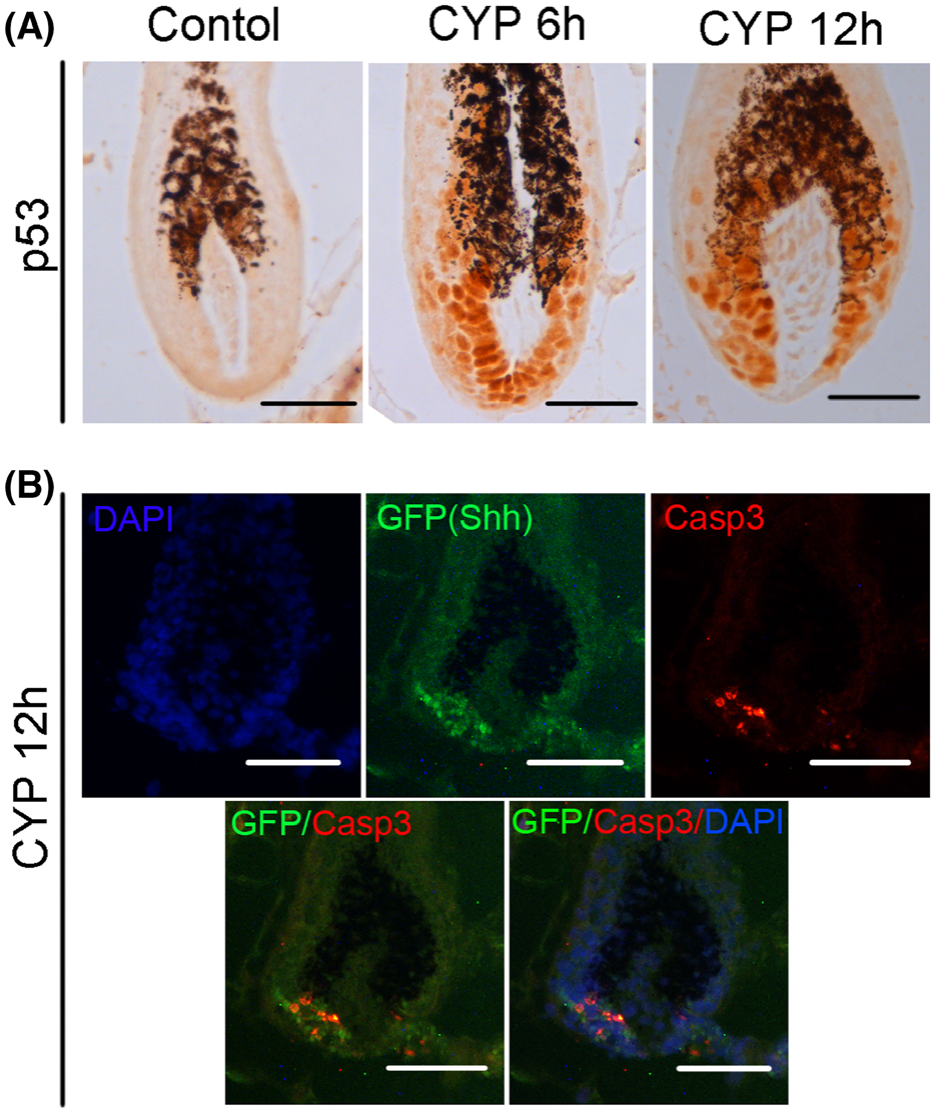
p53 and caspase‐3 were activated by CYP. (A) Immunohistochemistry staining of p53 in hair follicles of Shh‐GFP^+/−^ mice at 6 and 12 h post‐CYP treatment (scale bar = 50 μm). The p53 antibody was diluted at 1:200, and incubated at 4 °C overnight. Note almost the matrix cells of hair follicles were positive stained at 6 and 12 h post‐CYP treatment. (B) Immunofluorescence staining of active caspase‐3 in hair follicles of Shh‐GFP^+/−^ mice at 12 h post‐CYP treatment. The active caspase‐3 antibody was diluted at 1 : 200 and incubated at 4 °C overnight. Note the caspase‐3 (CY3) signal overlapped with the GFP signal (scale bar = 50 μm).

In accordance with this result, immunostaining of p53 in hair follicles revealed that p53 was obviously activated in the matrix cells, including Shh^+^ cells, of hair follicles both at 6 and 12 h post‐CYP treatment (Fig. 4A). Moreover, the active caspase‐3 signal (CY3, red) was found to overlap with the GFP signal at 12 h post‐CYP treatment (Fig. 4B). These results suggested that apoptosis, caused by the activation of p53, occurred in Shh^+^ cells during CIA, which may explain the decrease in Shh^+^ cells in hair follicles upon CYP treatment.

### The JAK/STAT1 signaling pathway participates in perturbing the expression of Shh

**Fig. 5:**
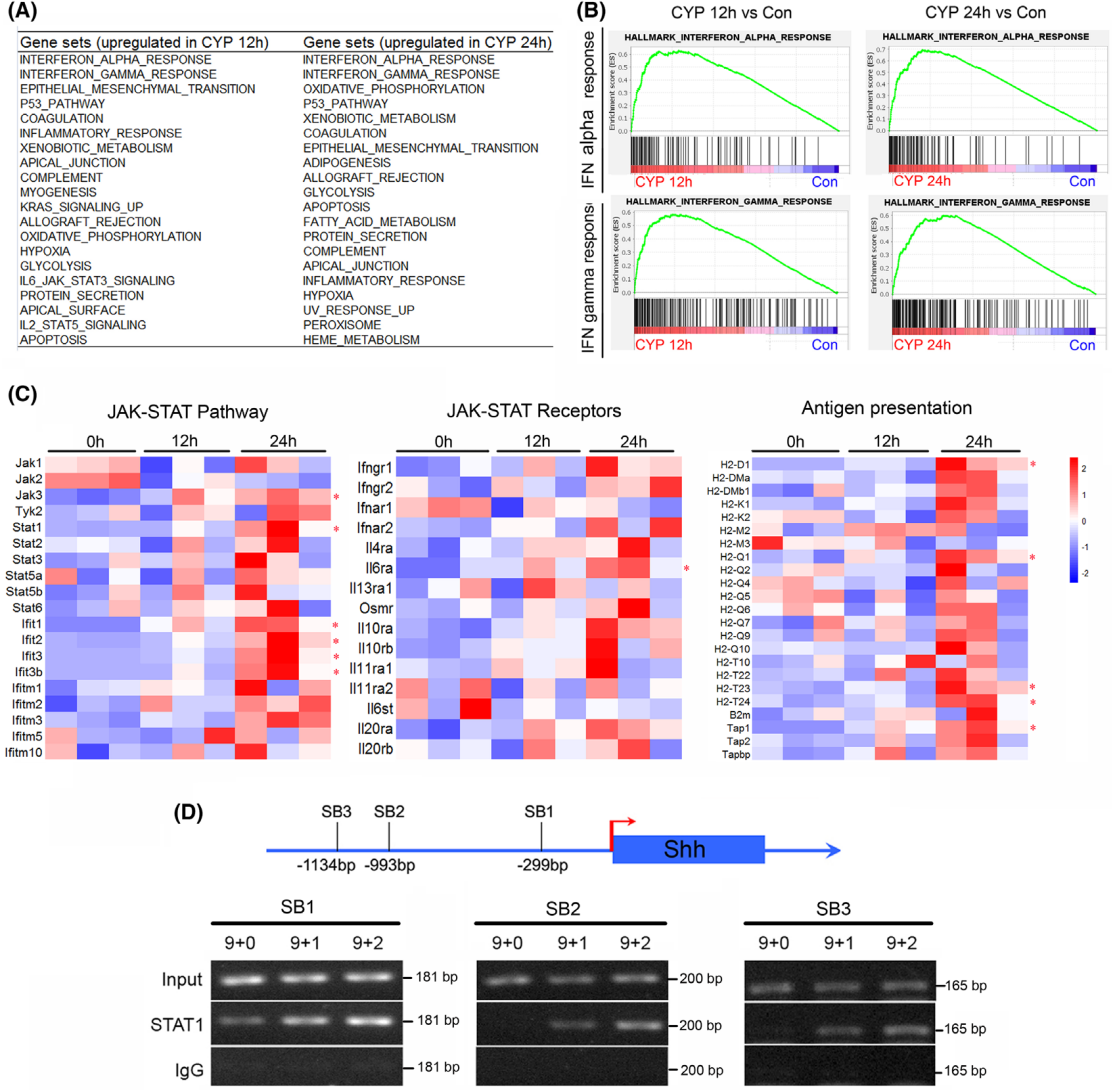
The JAK/STAT1 signaling pathway participated in the regulation of the expression of Shh. (A) Top 20 gene sets enriched in the CYP 12 h and CYP 24 h groups compared to the control group, respectively. (B) Enrichment plot of the gene set of the interferon gamma response at 12 and 24 h after CYP injection compared to the control group, respectively. (C) The expression patterns of genes related to JAK/STAT pathway, JAK/STAT receptors and antigen presentation. Student's *t*‐test was used to calculate the *P*‐value of mRNA levels of genes at CYP 24 h group compared to the 0 h group. *Significant difference determined at *P* < 0.05 and log_2_(fold change) ≥ 1 or log_2_(fold change)  ≤ −1. (D) Results of the ChIP PCR assay using STAT1 antibody. The input DNA had been diluted with Tris‐EDTA buffer at a ratio of 1 : 4 before PCR. The binding signals of all of the three tested STAT1 binding sites (SB1, SB2 and SB3) increased post‐CYP treatment, of which the SB1 binding site had the strongest signal.

Because KEGG pathway enrichment analysis focuses only on differentially expressed genes, it may miss some important signaling pathways that just have a few differentially expressed genes. To avoid this defect, GSEA was conducted to reveal more information in the RNA sequencing data using the mouse hallmark gene set database [18]. The results of GSEA showed that no gene set was significantly enriched in the control group compared to the CYP 12 h group or the CYP 24 h group according to the criteria (*P* < 0.05 and false discovery rate < 0.25). By contrast, there were 37 and 35 gene sets significantly enriched in the CYP 12 h group and the CYP 24 h group, respectively (Fig. 5A; Tables S6 and S7).

Interestingly, except for the p53 and apoptosis signaling pathways, two gene sets, such as the interferon alpha response and the interferon gamma response, were on top of the lists of drug‐perturbed gene sets at 12 and 24 h post‐CYP treatment (Fig. 5A,B). The interferons transmit signals through the classic JAK/STATs signaling pathway. To investigate the specific changes in the JAK/STAT signaling pathway, we compared the expression levels of genes related to JAK/STAT signal transduction, JAK/STAT pathway receptors and antigen presentation as described in a previous study [12]. The results showed that the expression levels of most JAK/STAT signaling pathway‐related genes exhibited an upward trend. Among the genes associated with JAK/STAT signal transduction, JAK3 and STAT1, as well as the interferon‐stimulated genes, including those for Ifit1, Ifit2, Ifit3 and Ifit3b, were significantly upregulated (Fig. 5C). Only Il6ra showed a significant increase among the JAK/STAT pathway receptors (Fig. 5C). In antigen presentation‐related genes, those for B2m, H2‐M3, H2‐T22, H2‐T23 and Tap1 were all significantly upregulated (Fig. 5C). Given the critical role of STAT proteins in the JAK/STAT signaling pathway, the high expression of Stat1 suggests that it may be a key factor mediating JAK signal transduction in CIA.

A previous report had demonstrated that STAT1 could directly bind to the promoter of the Shh gene and modify the expression of Shh in neuronal precursor cells [19]. To verify whether STAT1 could bind to the promoter of the Shh gene during CIA in the hair follicles, a ChIP assay was conducted and three candidate binding sites as described in the previous report [19] were investigated. The results showed that the binding signals of STAT1 on the three candidate binding sites all increased upon the CYP treatment (Fig. 5D and Fig. S2). This finding suggested that STAT1 may directly regulate the expression of Shh during CIA.

### JAK inhibitors increase Shh^+^ cells and up‐regulate the expression level of Shh during CIA

To further confirm the effect of the JAK/STAT signaling pathway on chemotherapy‐induced hair loss and the Shh gene, we investigated the impact of three JAK inhibitors, including AG490, ruxolitinib and tofacitinib, on chemotherapy‐induced alopecia. The experimental results showed that topical application of 1% AG490 and 1% ruxolitinib for five consecutive days (from day 7 to day 11 after depilation) rescued hair loss in mice, with treated mice rapidly growing a significant amount of gray hair on their backs. However, when the concentration was increased to 3%, no therapeutic effect was observed. By contrast, 1% tofacitinib showed no therapeutic effect, and it only exhibited a mild rescue effect when the concentration was increased to 3%, with a small amount of white hair growing at the edges of the treated mice's backs (*n* = 3 for each treatment) (Fig. S3).

**Fig. 6:**
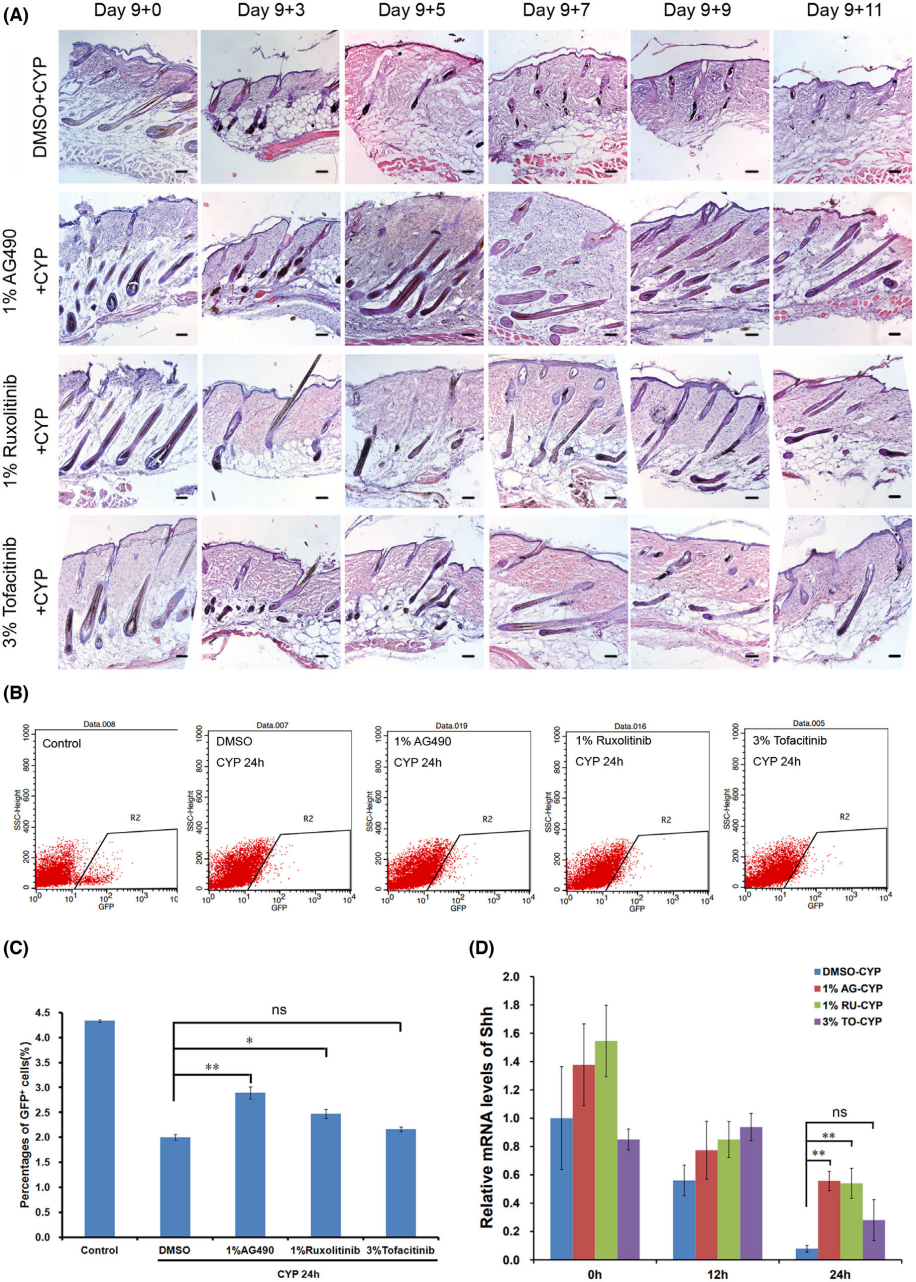
JAK inhibitors increased Shh^+^ cells and the expression levels of Shh. (A) H&E staining of whole skin samples of mice topically treated with 100 μL of dimethylsulfoxide or 100 μL of 1% AG490, 1% ruxolitinib and 3% tofacitinib, respectively, at different time points. Dimethylsulfoxide and the three JAK inhibitors were treated for 5 days from day 7 to day 11 after depilation. CYP was injected into mice on day 9 after depilation (scale bar = 100 μm). (B) Shh^+^ cells of hair follicle were analysis from Shh‐GFP^+/−^ mice treated with JAK inhibitors and CYP by flow cytometry before or 24 h after CYP treatment. (C) The differences of percentages of Shh^+^ cells among the treatments of different JAK inhibitor during CIA. The percentages of Shh^+^ cells in different treatments were compared with the dimethylsulfoxide group, respectively. The results are shown as the mean ± SEM. A two‐tailed Student's *t*‐test was used to calculate the *P*‐value (*n* = 3; *P < 0.05; **P < 0.01; ns, no significance). (D) The relative mRNA levels of Shh in whole skin samples of mice treated with different JAK inhibitors. The mRNA levels of Shh in different treatments were compared with the dimethylsulfoxide group, respectively, at different times post‐CYP treatment. The results are shown as the mean ± SEM. A two‐tailed Student's *t*‐test was used to calculate the *P*‐value. Significant differences were only observed at 24 h after CYP treatment (*n* = 3; *P < 0.05; **P < 0.01; ns, no significance).

H&E staining of mouse skin samples revealed that, in the control group, hair follicles were significantly damaged and atrophied by day 3 after CYP injection, and, by day 7, no hair follicles remained in the skin. By contrast, mice treated with JAK inhibitors exhibited a clear process of damage repair: hair follicles also showed damage and atrophy on day 3 after CYP injection, but, by day 5, almost intact hair follicles were detected and persisted throughout the observation period (Fig. 6A).

Flow cytometry analysis showed that 1% AG490 and 1% ruxolitinib significantly increased the number of Shh^+^ cells in hair follicles 24 h after CYP injection, whereas 3% tofacitinib only slightly increased the number of Shh^+^ cells, although the difference was not statistically significant (Fig. 6B,C). Similarly, quantitative PCR results indicated that 1% AG490 and 1% ruxolitinib significantly upregulated Shh gene expression in the skin 24 h after CYP injection, whereas 3% tofacitinib only slightly increased Shh expression, with no significant difference (Fig. 6D).

These results suggest that JAK inhibitors can increase the number of Shh^+^ cells and Shh gene expression in hair follicles, thereby rescuing chemotherapy‐induced hair loss. However, the efficacy of different inhibitors varied. Notably, 1% AG490 and 1% ruxolitinib were able to enhance Shh expression even at 0 h (before the treatment of CYP), which aligns with previous findings that JAK inhibitors can upregulate Shh expression [11].

## Discussion

Sonic hedgehog signaling is essential for the morphogenesis and cycling of hair follicles [20, 21, 22]. In previous studies, we found that the expression level of Shh significantly decreased post‐CYP treatment, demonstrating that the Shh signaling pathway is an important target of chemo‐drugs [7, 8]. However, how chemo‐drugs suppress the expression of Shh remains unknown. As a result of the localized expression of Shh and the small amount of Shh^+^ cells in hair follicles, studies using whole skin samples may be biased. Non‐Shh‐expressing components of skin may strongly interfere in exploring the mechanism of the regulation of Shh during CIA.

To avoid this defect, the Shh‐GFP^+/−^ mouse was used in the present study. The heterozygote of the transgenic mouse was first found to undergo CIA, as well as the wild‐type mouse, regardless of gender, with similar phenotypes and hair follicle defects (Fig. 1). The percentage of Shh^+^ cells decreased significantly at both 12 and 24 h post‐CYP treatment (Fig. 2). Importantly, this explains the decrease in mRNA and protein levels of Shh in a previous study [8]. The results of KEGG enrichment analysis of the RNA sequencing data revealed that the p53 signaling pathway and the apoptosis signaling pathway were significantly enriched in Shh^+^ cells at both 12 and 24 h after CYP injection (Fig. 3D; Tables S4 and S5). In line with this finding, p53 and caspase‐3 were found to be activated in Shh^+^ cells post‐CYP treatment by immunostaining (Fig. 4). These results suggest that the decrease in Shh^+^ cells in hair follicles during CIA was mainly a result of apoptosis.

Notably, except for the decrease in Shh^+^ cells, the mRNA level of Shh in Shh^+^ cells also decreased significantly (Fig. 3C). This strongly suggests that the reduction of Shh in skin or hair follicles is a result not only of the reduction of Shh^+^ cells, but also the down‐regulation of Shh. Interestingly, using GSEA analysis, more gene sets were found, of which the interferon alpha response and the interferon gamma response were on the top lists of enriched gene sets at both 12 and 24 h post‐CYP treatment (Fig. 5A,B; Tables S6 and S7). Moreover, the downstream transductor of interferons, STAT1, was found to bind to the promoter of Shh at three different sites along with CYP treatment (Fig. 5D). This result suggests that STAT1 may directly regulate the expression of Shh during CIA.

Although the JAK/STAT1 signaling pathway has been confirmed to stimulate the expression of Shh in other cells or tissues, such as neuronal precursor cells [19], breast cancer cells [23] and the brain [24], inhibiting JAK/STAT1 signaling pathway using JAK inhibitors in the present study resulted in rescuing CIA in mice (Fig. S3) and increasing Shh+ cells, in addition to the expression level of Shh in hair follicles (Fig. 6). This result suggests the regulation of Shh by the JAK/STAT1 signaling pathway in mouse hair follicles during CIA may be different from other conditions, or that some other signaling pathways may be involved to regulate the expression of Shh. These extrinsic signaling pathways may originate from dermal papilla cells because a preliminary study [11] found that JAK inhibitors can activate the function of dermal papilla to promote Shh expression, thereby facilitating hair follicle entry into the growth cycle. Because the regulation of Shh gene expression is very complex, with many specific enhancers involved [25, 26], more work will be required to reveal the molecular mechanism of the down‐regulation of Shh during CIA.

Interestingly, a previous study has shown that the long‐term use of JAK inhibitors can promote hair regeneration by reconstructing the hair follicle stem cell (HFSC) niche and facilitating the redevelopment of HFSCs [12]. This holds significant value for the treatment of conditions such as scarring alopecia. However, chemotherapy‐induced hair loss involves a rapid and severe injury process to hair follicles, which differs from the pathogenesis of scarring alopecia. The present study reveals that JAK inhibitors can promote the rapid repair of damaged hair follicles within approximately 2 days, restoring intact hair follicle structures, comprising a mechanism distinct from the *de novo* development of HFSCs. This effect may be associated with the enhancement of Shh^+^ cells and Shh expression because Shh can promote cell proliferation of hair follicle [20]. Because the present study did not examine the status of dermal papilla cells, the therapeutic effect of JAK inhibitors on chemotherapy‐induced hair loss cannot exclude the potential influences of dermal papilla cells [11].

Furthermore, the present study found varying efficacy among different JAK inhibitors with respect to treating chemotherapy‐induced alopecia, with the effectiveness ranking as 1% AG490 > 1% ruxolitinib > 3% tofacitinib. Because these inhibitors target different kinases, with AG490 primarily inhibiting JAK2 [27, 28], ruxolitinib mainly targeting JAK1/JAK2 [29] and tofacitinib predominantly inhibiting JAK1/JAK3 [29], the respective effectiveness of the different JAK inhibitors suggests that JAK2 may be a critical target for CIA treatment. However, this observation appears inconsistent with the RNA sequencing results from the present study, which showed only JAK3 was significantly upregulated after CYP injection. A potential explanation for this discrepancy lies in the nature of these inhibitors as protein kinase inhibitors, which directly affect JAK kinase activation and function. The protein level and phosphorylation status of intracellular JAK kinases are the key determinants of signal transduction. In the present study, raw mRNA expression data (fragments per kilobase of transcript per million mapped reads) of JAK kinases revealed that JAK2 expression was approximately 18 times higher than that of JAK3 before CYP treatment (0 h) and, even 24 h after CYP treatment, JAK2 expression remained 2.4 times higher than JAK3 (Table S8). These findings suggest that the protein level of JAK2 in Shh^+^ cells is likely higher than that of JAK3. Therefore, determining which specific kinase plays the dominant role would require further investigation into the protein levels and the phosphorylation status of JAK kinases.

In conclusion, the results of the present study have shown that Shh^+^ cells in hair follicles decreased significantly post‐CYP treatment, which may be a result of apoptosis in these cells caused by the activated p53 signaling pathway and the apoptosis signaling pathway. Moreover, the expression of the Shh gene also decreased obviously in Shh^+^ cells, and the JAK/STAT1 signaling pathway participated in the downregulation of Shh during CIA. The findings of the present study also suggested that inhibitors of the JAK/STAT1 signaling pathway may comprise new candidate drugs for preventing CIA.

## Conflicts of interest

The authors declare that they have no conflicts of interest.

## Author contributions

RF, JH TL, YT, LS and GZ performed the experiments. RF, JH and GZ analyzed the data. RF and GZ interpreted the data. RF and GZ wrote the manuscript. XL analyzed the RNA sequencing data and revised the manuscript. GZ designed the study and wrote the manuscript. All authors have read and approved the final version of the manuscript submitted for publication.

## Supporting information


**Fig. S1.** Genotyping of Shh‐GFP mice.

**Fig. S2.** Uncropped DNA gels of ChIP‐PCR.

**Fig. S3.** JAK inhibitors rescue hair loss on the dorsal skin of mice during CIA.

**Table S1.** List of primers used in the present study.

**Table S2.** List of differentially expressed genes (CYP 12 h versus Control).

**Table S3.** List of differentially expressed genes (CYP 24 h versus Control).

**Table S4.** List of KEGG pathways enrichment result (CYP 12 h versus Control).

**Table S5.** List of KEGG pathways enrichment result (CYP 24 h versus Control).

**Table S6.** List of GSEA result (CYP 12 h versus Control).

**Table S7.** List of GSEA result(CYP 24 h versus Control).

**Table S8.** The raw mRNA expression data (fragments per kilobase of transcript per million mapped reads) of different JAK kinases.

## Data Availability

The RNA sequencing raw data has been deposited in the NCBI GEO database (accession number: GSE277257).
