# Supplementary figures and images for: Down‐regulation of Shh in the hair follicles of mice during chemotherapy‐induced hair loss is mediated by the JAK/STAT1 signaling pathway
Source: FEBS Open Bio. 2025 Nov 20;16(5):966–78. doi: 10.1002/2211-5463.70160 (PMC13145336; doi:10.1002/2211-5463.70160)

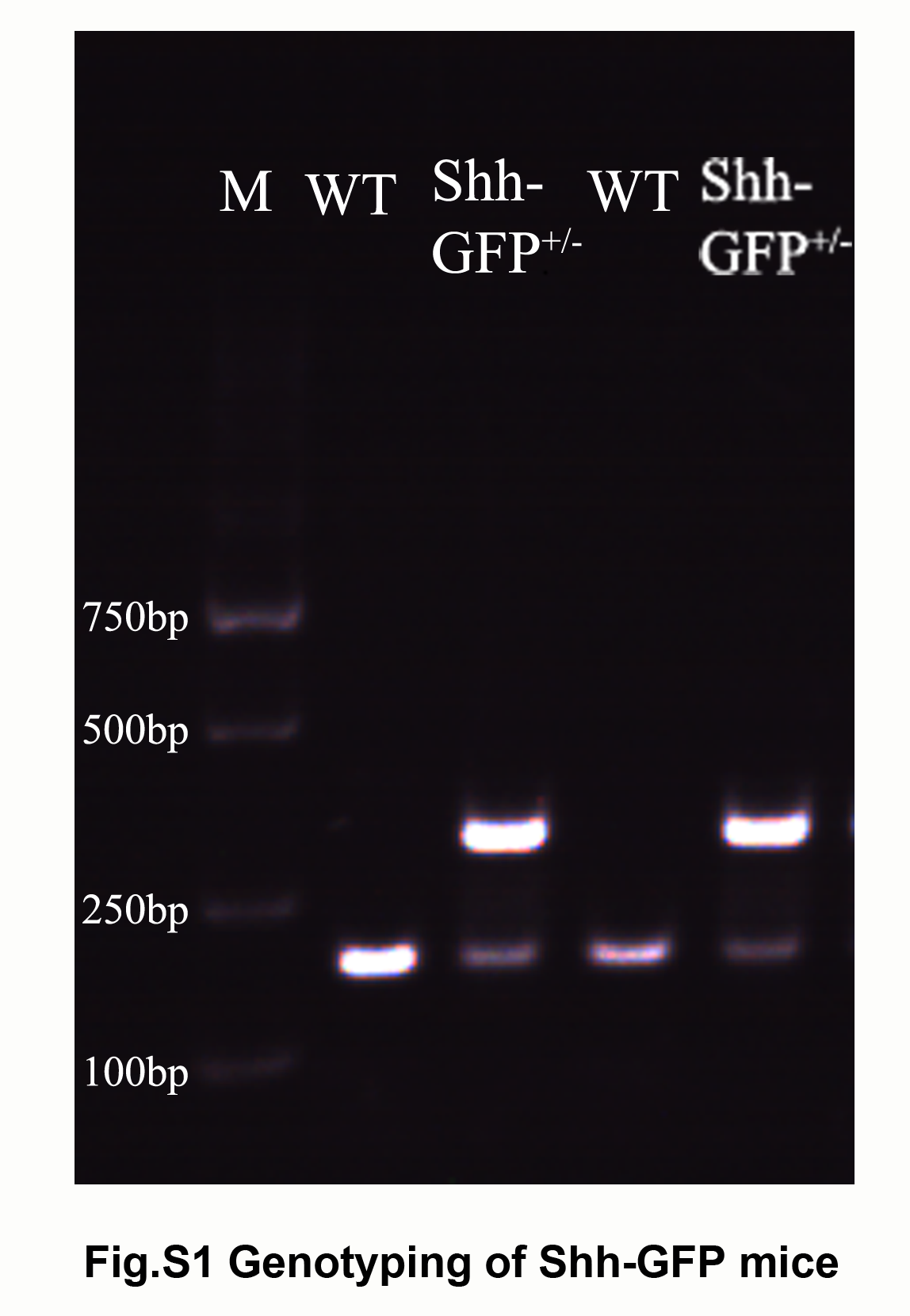

Supplement: Supplementary file 1 — Fig. S1. Genotyping of Shh‐GFP mice. [file FEB4-16-966-s003.tiff]

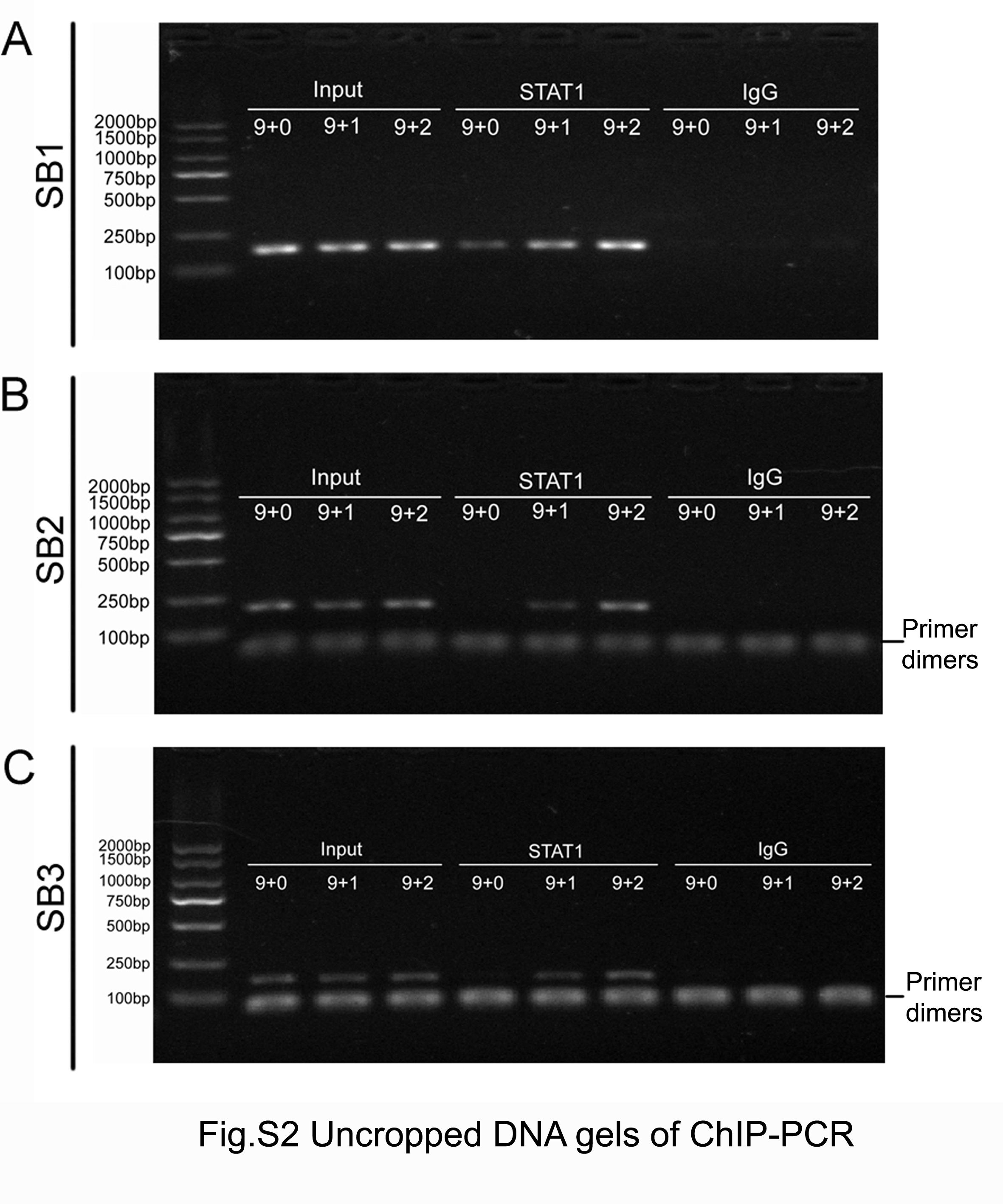

Supplement: Supplementary file 2 — Fig. S2. Uncropped DNA gels of ChIP‐PCR. [file FEB4-16-966-s008.tiff]

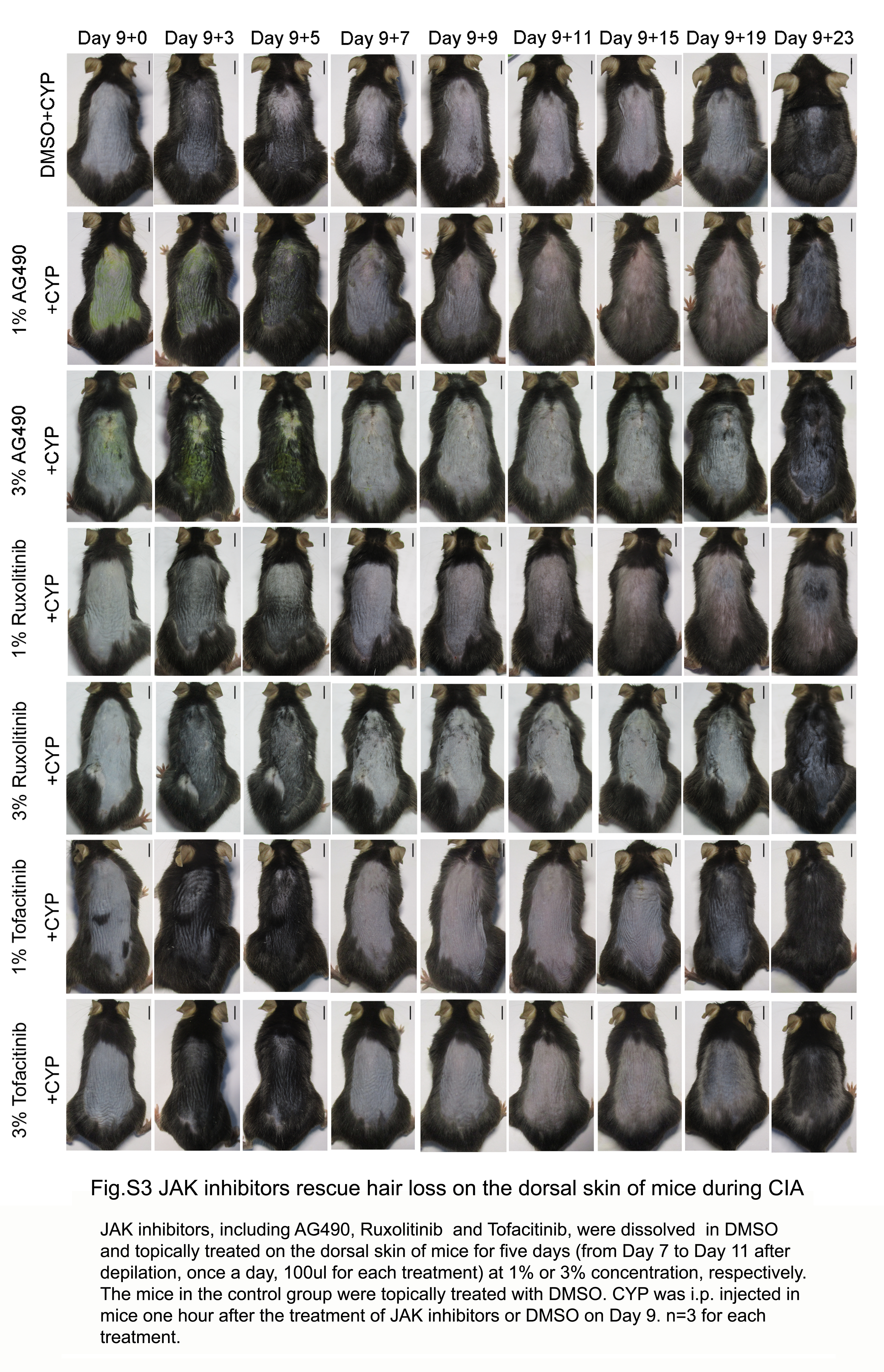

Supplement: Supplementary file 3 — Fig. S3. JAK inhibitors rescue hair loss on the dorsal skin of mice during CIA. [file FEB4-16-966-s010.tif]
